# Gravitational Equivalence Theorem and Double-Copy for Kaluza-Klein Graviton Scattering Amplitudes

**DOI:** 10.34133/2022/9860945

**Published:** 2022-07-14

**Authors:** Yan-Feng Hang, Hong-Jian He

**Affiliations:** ^1^ Tsung-Dao Lee Institute and School of Physics and Astronomy, Key Laboratory for Particle Astrophysics and Cosmology (MOE), Shanghai Key Laboratory for Particle Physics and Cosmology, Shanghai Jiao Tong University, Shanghai, China; ^2^ Institute of Modern Physics and Department of Physics, Tsinghua University, Beijing, China; ^3^ Center for High Energy Physics, Peking University, Beijing, China

## Abstract

We analyze the structure of scattering amplitudes of the Kaluza-Klein (KK) gravitons and of the KK gravitational Goldstone bosons in the compactified 5d general relativity (GR). Using a general $R_{\xi }$ gauge fixing, we study the geometric Higgs mechanism for the massive spin-2 KK gravitons. We newly propose and prove a gravitational equivalence theorem (GRET) to connect the scattering amplitudes of longitudinal KK gravitons to that of the KK gravitational Goldstone bosons, which *formulates the geometric gravitational Higgs mechanism at the scattering* S - *matrix level.* We demonstrate that *the GRET provides a general energy cancellation mechanism* guaranteeing the N -point longitudinal KK graviton scattering amplitudes to have their leading energy dependence cancelled down by a large power factor of $E^{2N}\left(N\geq 4\right)$ up to any loop level. We propose an improved double-copy approach to construct the massive KK graviton (Goldstone) amplitudes from the KK gauge boson (Goldstone) amplitudes. With these, we establish *a new correspondence between the two types of energy cancellations* in the four-point longitudinal KK amplitudes at tree level: $E^{4}⟶E^{0}$ in the KK gauge theory and $E^{10}⟶E^{2}$ in the KK GR theory.

## 1. Introduction

Kaluza-Klein (KK) compactification [ 1, 2] of the extra spatial dimensions leads to infinite towers of massive KK excitation states in the low-energy 4d effective field theory. This serves as an essential ingredient of all extra dimensional models [ 3– 5] and the string/ M theories [ 6]. The KK compactification realizes the geometric “Higgs” mechanisms for mass generations of KK gravitons [ 7, 8] and of KK gauge bosons [ 9] without invoking any extra Higgs boson of the conventional Higgs mechanism [ 10– 14].

In this work, we formulate the geometric gravitational “Higgs” mechanism for the compactified 5d general relativity (GR5) by quantizing the KK GR5 under a general $R_{\xi }$ gauge fixing at both the Lagrangian level and the S -matrix level. We prove that the KK graviton propagator is *free from* the longstanding problem of van Dam-Veltman and Zakharov (vDVZ) discontinuity [ 15, 16] in the conventional Fierz-Pauli massive gravity [ 17, 18] and the KK GR5 theory can *consistently realize the mass generation for spin-2 KK gravitons.* Then, we propose and prove a new gravitational equivalence theorem (GRET) which quantitatively connects each scattering amplitude of the (helicity-zero) longitudinally polarized KK gravitons to that of the corresponding KK Goldstone bosons. The GRET takes a highly nontrivial form and differs substantially from the KK gauge equivalence theorem (GAET) of the 5d KK gauge theories [ 9, 19, 20], because each massive KK graviton $h_{n}^{\mu \nu }$ has 5 helicity states ( λ=0 , ±1, ±2) where the λ=0 , ±1 components arise from absorbing a scalar Goldstone boson $h_{n}^{55}\left(\lambda =0\right)$ and a vector Goldstone boson $h_{n}^{\mu 5}\left(\lambda =\pm 1\right)$ in the 5d graviton field. We demonstrate that *the GRET provides a general energy cancellation mechanism* guaranteeing that the leading energy dependence of N -particle longitudinal KK graviton amplitudes $\left(\propto E^{2\left(N+1+L\right)}\right)$ must cancel down to a much lower energy power $\left(\propto E^{2\left(1+L\right)}\right)$ by an energy factor of $E^{2N}$ , as enforced by matching the energy dependence of the corresponding leading gravitational KK Goldstone amplitudes, where L denotes the loop number of the relevant Feynman diagram. For the four-point longitudinal KK graviton scattering amplitudes at tree level, this proves the energy cancellations $E^{10}⟶E^{2}$ , which explains the result of the recent explicit calculations of 4-longitudinal KK graviton amplitudes [ 21– 23].

The double-copy approach has profound importance for understanding the quantum gravity because it uncovers the deep gauge-gravity connection at the scattering S -matrix level, $\text{GR}={\left(\text{Gauge Theory}\right)}^{2}$ [ 24]. The conventional double-copy method with color-kinematics (CK) duality of Bern-Carrasco-Johansson (BCJ) [ 25– 27] was proposed to connect scattering amplitudes between the massless Yang-Mills (YM) gauge theories and the massless GR theories. It was inspired by the Kawai-Lewellen-Tye (KLT) relation [ 28] which connects the product of two scattering amplitudes of open strings to that of the closed string at tree level [ 29].

Extending the conventional double-copy approach, we construct the massive KK graviton (Goldstone) amplitudes from the massive KK YM gauge (Goldstone) amplitudes under high energy expansion at the leading order (LO) and at the next-to-leading order (NLO). This provides an extremely efficient way to derive the complicated massive KK graviton amplitudes from the massive KK gauge boson amplitudes and gives a deep understanding on the structure of the KK graviton amplitudes.

Because the LO amplitudes of the longitudinal KK gauge bosons and of their KK Goldstone bosons have $O\left(E^{0}M_{n}^{0}\right)$ and are equal (leading to the KK GAET) [ 9], our double-copy approach demonstrates that the reconstructed LO amplitudes of the longitudinal KK gravitons and of the KK Goldstone bosons have $O\left(E^{2}M_{n}^{0}\right)$ and must be equal to each other (leading to the KK GRET), where $M_{n}$ denotes the relevant KK mass. Our double-copy construction further proves that the residual term of the GRET belongs to the NLO, which has $O\left(E^{0}M_{n}^{2}\right)$ and is suppressed by the factor $M_{n}^{2}/E^{2}$ relative to the LO KK Goldstone boson amplitude of $O\left(E^{2}M_{n}^{0}\right)$ .

## 2. $R_{\xi }$ Gauge Fixing and Geometric Higgs Mechanism

We consider the compactified GR5 under the orbifold $S^{1}/{\mathbb{Z}}_{2}$ where the fifth dimension is a line segment $0\leq x^{5}\leq L\left(=\pi r_{c}\right)$ , with $r_{c}$ being the compactification radius. Extension to the case of warped 5d space [ 5] does not cause conceptual change regarding our current study. Thus, the 5d Einstein-Hilbert (EH) action takes the following form:

(1)SEH=∫d5xL^EH=∫d5x2κ∧2−g^R^,

where the coupling constant $\hat{\kappa }=\sqrt{32\pi \hat{G}}$ .

Then, we expand the 5d EH action ( 1) under the metric perturbation ${\hat{\text{g}}}_{AB}={\hat{\eta }}_{AB}+\hat{\kappa }{\hat{h}}_{AB}$ , where ${\hat{\eta }}_{AB}=\mathrm{diag}\left(-1,1,1,1,1\right)$ is the 5d Minkowski metric. Thus, we can express the 5d graviton field ${\hat{h}}_{AB}$ as follows:

(2)h^AB=h^μν−12ημνϕ^h^μ5h^5νϕ^.

Under the compactification of $S^{1}/{\mathbb{Z}}_{2}$ , the spin-2 field ${\hat{h}}_{\mu \nu }$ and scalar field $\hat{\phi }\left(\equiv {\overset{\wedge }{h}}^{55}\right)$ are ${\mathbb{Z}}_{2}$ even, while the vector field ${\hat{A}}_{\mu }\left(\equiv {\hat{h}}_{\mu 5}\right)$ is ${\mathbb{Z}}_{2}$ odd. After compactification, we derive the 4d effective Lagrangian for both the zero modes and KK modes $\left(h_{n}^{\mu \nu },A_{n}^{\mu },\phi_{n}\right)$ [ 30].

We further construct a general $R_{\xi }$ -type gauge fixing term:

(3)LGF=−∑n=0∞1ξnFnμ2+Fn52,

where $\left(F_{n}^{\mu },F_{n}^{5}\right)$ take the following form [ 31]:

(4a)Fnμ=∂νhnμν−1−12ξn∂μhn+ξnMnAnμ,(4b)Fn5=12Mnhn−3ξnMnϕn+2∂μAnμ.

The above $R_{\xi }$ gauge fixing can ensure the kinetic terms and propagators of the KK fields $\left(h_{n}^{\mu \nu },A_{n}^{\mu },\phi_{n}\right)$ to be diagonal. In the limit of $\xi_{n}⟶\infty$ , we recover the unitary gauge where the KK Goldstone bosons $\left(A_{n}^{\mu },\phi_{n}\right)$ are fully absorbed (eaten) by the corresponding KK gravitons $h_{n}^{\mu \nu }$ at each KK level- n . This realizes a geometric gravitational “Higgs” mechanism for KK graviton mass generations.

Then, we derive the propagators of KK gravitons and KK Goldstone bosons under the $R_{\xi }$ gauge fixing ( 3) [ 30]. For Feynman-‘t Hooft gauge $\left(\xi_{n}=1\right)$ , the propagators take the following simple forms:

(5a)Dnmμναβp=−iδnm2ημαηνβ+ημβηνα−ημνηαβp2+Mn2,(5b)Dnmμνp=−iημνδnmp2+Mn2,Dnmp=−iδnmp2+Mn2,

which all share the same mass-pole $p^{2}=-M_{n}^{2}$ , with the KK mass $M_{n}=n\pi /L$ .

Strikingly, we observe that our massive KK graviton propagator ( 5a) has a smooth limit for $M_{n}⟶0$ , under which Equation ( 5a) reduces to the conventional massless graviton propagator of Einstein gravity. Hence, have proven that *the KK graviton propagator is free from the vDVZ discontinuity* [ 15, 16] which is a longstanding problem plaguing the conventional Fierz-Pauli massive gravity and alike [ 17, 18]. This is because the GHM under KK compactification guarantees that the physical degrees of freedom of each KK graviton are *conserved before and after taking the massless limit* $M_{n}⟶0$ , i.e., 5=2+2+1 . This demonstrates that the compactified KK GR can consistently realize the mass-generation for spin-2 KK gravitons.

## 3. GRET Formulation for the GHM

In the previous section, we analyzed the geometric Higgs mechanism at the Lagrangian level. In this section, we further formulate the GRET, which *realizes the geometric gravitational Higgs mechanism at the* S - *matrix level*. Using the gauge-fixing terms ( 3)-( 4) and following the method of Ref. [ 32, 33], we derive a Slavnov-Taylor-type identity in the momentum space:

(6)0Fn1μ1k1Fn2μ2k2⋯Fm15p1Fm25p2⋯Φ0=0,

where Φ denotes any other on-shell physical fields after the Lehmann-Symanzik-Zimmermann (LSZ) amputation and each external momentum obeys the on-shell condition $k_{j}^{2}=-M_{n_{j}}^{2}\text{or}p_{j}^{2}=-M_{m_{j}}^{2}$ . The identity ( 6) is a direct consequence of the diffeomorphism (gauge) invariance of the theory [ 31, 32].

Under the Feynman-‘t Hooft gauge $\left(\xi_{n}=1\right)$ and at the tree level, we can directly amputate each external state by multiplying the propagator-inverse $\left(k^{2}+M_{n}^{2}\right)⟶0$ for Equation ( 6). Thus, we derive [ 31] the following GRET identity which connects the longitudinal KK graviton amplitude to the corresponding KK Goldstone amplitude plus a residual term:

(7a)Mhn1L,⋯,hnNL,Φ=Mϕn1,⋯,ϕnN,Φ+MΔ,(7b)MΔ=∑1≤k≤NMΔ~nk,ϕn,Φ,

where ${\tilde{\Delta }}_{n}={\tilde{v}}_{n}-{\tilde{h}}_{n},{\tilde{v}}_{n}={\tilde{v}}_{\mu \nu }h_{n}^{\mu \nu }$ and ${\tilde{h}}_{n}=\sqrt{2/3}\eta_{\mu \nu }h_{n}^{\mu \nu }$ . The tensor ${\tilde{v}}^{\mu \nu }=\varepsilon_{L}^{\mu \nu }-\sqrt{2/3}\varepsilon_{S}^{\mu \nu }=O\left(E^{0}\right)$ and $\left(\varepsilon_{L}^{\mu \nu },\varepsilon_{S}^{\mu \nu }\right)$ are the (longitudinal, scalar) polarizations of the KK graviton $h_{n}^{\mu \nu }$ . We can extend the GRET ( 7) up to loop levels and valid for all $R_{\xi }$ gauges by using the gravitational BRST identities [ 34], similar to the ET formulation in the 5d KK YM theories [ 21] and in the 4d SM [ 32, 33, 35, 36].

Inspecting the scattering amplitudes in the GRET identity ( 7a), we can make direct power counting on the leading E -dependence of individual Feynman diagrams for each amplitude. For the 4-particle scattering, the longitudinal KK graviton amplitude on the left-hand-side of Equation ( 7a) contains individual contributions via quartic interactions or via exchanging KK-mode (zero-mode) gravitons. Since each external longitudinal KK graviton has polarization tensor $\varepsilon_{L}^{\mu \nu }⊃k^{\mu }k^{\nu }/M_{n}^{2}$ , the leading individual contributions behave as $O\left(E^{10}\right)$ . But we observe that on the right-hand-side (RHS) of Equation ( 7a), the external states in all amplitudes have no superficial enhancement or suppression factor. Thus, by power counting on the KK amplitudes, we find that the RHS of Equation ( 7a) (including the residual term $M_{\Delta }$ ) scales as $O\left(E^{2}\right)$ . Hence, the GRET identity ( 7) provides *a general mechanism* for the large energy cancellations of $E^{10}⟶E^{2}$ in the 4-longitudinal KK graviton amplitudes.

We have further developed a generalized energy-power counting method [ 30] for the massive KK gauge and gravity theories, by extending the conventional 4d power counting rule of Steven Weinberg for the nonlinear sigma model of low energy QCD [ 37, 38]. With this and the GRET ( 7), we can prove a general energy cancellation $E^{2\left(N+1+L\right)}⟶E^{2\left(1+L\right)}$ in the N -point longitudinal KK graviton amplitudes, which cancels the leading energy-dependence by $E^{2N}$ powers [ 30]. For N -longitudinal KK gauge boson amplitudes, we also prove [ 31] a general energy cancellation of $E^{4}⟶E^{4-N-\delta }$ , which cancels the leading E -powers by $E^{N+\delta }$ , with $\delta =\left[1-{\left(-1\right)}^{N}\right]/2$ . We will establish *a new correspondence* between the two types of energy cancellations in the N -point KK gauge boson amplitudes and KK graviton amplitudes in Section 5.

## 4. KK Graviton Scattering Amplitudes from GRET

**Figure 1:**
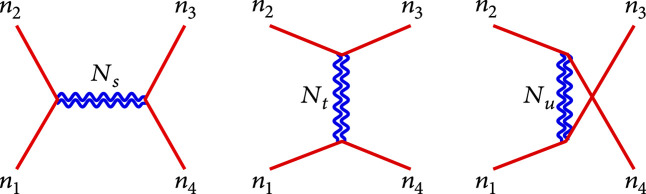
Feynman diagrams for the scattering of gravitational KK Goldstone bosons, $\phi_{n_{1}}\phi_{n_{2}}⟶\phi_{n_{3}}\phi_{n_{4}}$ , by exchanging the KK gravitons of level- $N_{j}\left(j=s,t,u\right)$ at the tree level, which contribute the leading energy-dependence of $O\left(E^{2}\right)$ .

In the following, we demonstrate explicitly how the GRET holds. For this, we compute the gravitational KK Goldstone boson scattering amplitude $\tilde{M}\left[\phi_{n_{1}}\phi_{n_{2}}⟶\phi_{n_{3}}\phi_{n_{4}}\right]\left(n_{j}\geq 1\right)$ . The relevant Feynman diagrams having leading energy contributions are shown in Figure 1.

For the elastic scattering, we set the KK numbers of all external states as $n_{i}=n$ and of internal states as $N_{j}=0,2n$ . Then, summing up the contributions of Figure 1 and making high energy expansion, we derive the following LO scattering amplitude of the gravitational KK Goldstone bosons:

(8)M~0=3κ21287+cos2θ2sin2θs,

where ${\tilde{M}}_{0}={\tilde{M}}_{0}\left[\phi_{n}\phi_{n}⟶\phi_{n}\phi_{n}\right]$ . To compare our Equations ( 8) with the corresponding longitudinal KK graviton amplitude of Refs. [ 21, 22], we rescale our coupling $\kappa ⟶\kappa /\sqrt{2}$ to match their normalization and find that *the two amplitudes are equal at the LO*:

(9)M0hLnhLn⟶hLnhLn=M~0ϕnϕn⟶ϕnϕn.

Namely, $M_{0}={\tilde{M}}_{0}$ , where we denote $M_{0}=M_{0}\left[h_{L}^{n}h_{L}^{n}⟶h_{L}^{n}h_{L}^{n}\right]$ and ${\tilde{M}}_{0}={\tilde{M}}_{0}\left[\phi_{n}\phi_{n}⟶\phi_{n}\phi_{n}\right]$ . From the GRET identity ( 7a) (and Equation ( 18)), this means that the residual term ( 7b) belongs to the NLO:

(10)MΔ=M−M~=δM−δM~=OE0Mn2,

and thus is much smaller. We have further computed the exact tree-level Goldstone boson amplitude $\tilde{M}$ by including all the subleading diagrams [ 30].

For inelastic scattering of gravitational KK Goldstone bosons, we compute the 4-point amplitudes and find that the LO inelastic amplitude is connected to the LO elastic amplitude ( 8) by

(11)M~ϕn1ϕn2⟶ϕn3ϕn4=ζM~ϕnϕn⟶ϕnϕn,

where ζ=2/3 for $n_{1}=n_{2}\neq n_{3}=n_{4}$ , and ζ=1/3 for the cases with KK numbers $\left(n_{1},n_{2},n_{3},n_{4}\right)$ having no more than one equality.

## 5. Double-Copy Construction of KK Amplitudes

The double-copy construction for the massive KK gauge/gravity scattering amplitudes are highly nontrivial. We make the first serious attempt for an explicit double-copy construction of KK amplitudes under high energy expansion. We present the 4-point elastic scattering amplitudes of longitudinal KK gauge bosons (Goldstones) at the LO and NLO:

(12a)T=∑jg2CjNjsj=∑jg2CjNj0+δNjsj=T0+δT,(12b)T~=∑jg2CjN~jsj=∑jg2CjN~j0+δN~jsj=T~0+δT~,

where we have denoted $T\equiv T\left[A_{L}^{\text{an}}A_{L}^{bn}⟶A_{L}^{cn}A_{L}^{dn}\right]$ and $\tilde{T}\equiv \tilde{T}\left[A_{5}^{\text{an}}A_{5}^{bn}⟶A_{5}^{cn}A_{5}^{dn}\right]$ . We also define the SU $\left(N\right)$ color factors as $\left(C_{s},C_{t},C_{u}\right)\equiv \left(C^{abe}C^{cde},C^{ade}C^{bce},C^{ace}C^{dbe}\right)$ , which obey the Jacobi identity $C_{s}+C_{t}+C_{u}=0$ .

We present in Table 1 the numerator factors $\left(N_{j},{\tilde{N}}_{j}\right)$ of Equations ( 12a) and ( 12b). Table 1 shows that $\left(T_{0},{\tilde{T}}_{0}\right)=O\left(E^{0}M_{n}^{0}\right)$ and $\left(\delta T,\delta \tilde{T}\right)=O\left(M_{n}^{2}/E^{2}\right)$ . We find that the sum of each set of the LO, NLO, and NNLO numerators of the KK gauge (Goldstone) scattering amplitudes in Equations ( 12) violate the kinematic Jacobi identity by terms of $O\left(M_{n}^{2}\right)$ and $O\left(M_{n}^{4}/E^{2}\right)$ , respectively:

(13a)∑jNj0=10cθMn2,∑jN~j0=−6cθMn2,(13b)∑jδ1Nj=∑jδ1N~j=−27+c2θcθcsc2θMn2,(13c)∑jδ2Nj=831+c4θcθcsc4θMn4s,(13d)∑jδ2N~j=327+c2θcθcsc4θMn4s,

where $c_{n\theta }=\cos\left(n\theta \right)$ , $\delta N_{j}=\delta_{1}N_{j}+\delta_{2}N_{j}$ , and $\delta {\tilde{N}}_{j}=\delta_{1}{\tilde{N}}_{j}+\delta_{2}{\tilde{N}}_{j}$ . Hence, we cannot naively apply color-kinematics duality for BCJ-type double-copy construction without making further modifications on these numerators.

**Table 1:** Kinematic numerators of the LO and NLO scattering amplitudes for KK longitudinal gauge bosons and KK Goldstones as defined in Equation ( 12), where $\left(N_{j},{\tilde{N}}_{j}\right)=\left(N_{j}^{0},{\tilde{N}}_{j}^{0}\right)+\left(\delta N_{j},\delta {\tilde{N}}_{j}\right)=O\left(E^{2}M_{n}^{0}\right)+O\left(E^{0}M_{n}^{2}\right)$ and $\left(s_{\theta },c_{\theta }\right)=\left(\sin\theta ,\cos\theta \right)$ .

Numerators	$N_{s}$	$N_{t}$	$N_{u}$	${\tilde{N}}_{s}$	${\tilde{N}}_{t}$	${\tilde{N}}_{u}$	$N_{s}-{\tilde{N}}_{s}$	$N_{t}-{\tilde{N}}_{t}$	$N_{u}-{\tilde{N}}_{u}$
$\frac{N_{j}^{0}}{s_{j}}$	$\frac{5c_{\theta }}{2}$	$\frac{13+5c_{\theta }+4c_{2\theta }}{2\left(1+c_{\theta }\right)}$	$-\frac{13-5c_{\theta }+4c_{2\theta }}{2\left(1-c_{\theta }\right)}$	$-\frac{3c_{\theta }}{2}$	$\frac{3\left(3-c_{\theta }\right)}{2\left(1+c_{\theta }\right)}$	$-\frac{3\left(3+c_{\theta }\right)}{2\left(1-c_{\theta }\right)}$	$4c_{\theta }$	$4c_{\theta }$	$4c_{\theta }$
$\frac{\delta N_{j}}{M_{n}^{2}}$	$4c_{\theta }$	$\frac{2\left(2-3c_{\theta }-2c_{2\theta }-c_{3\theta }\right)}{1+c_{\theta }}$	$-\frac{2\left(2+3c_{\theta }-2c_{2\theta }+c_{3\theta }\right)}{1-c_{\theta }}$	$4c_{\theta }$	$-\frac{8c_{\theta }}{1+c_{\theta }}$	$-\frac{8c_{\theta }}{1-c_{\theta }}$	0	$8s_{\theta }^{2}$	$-8s_{\theta }^{2}$

Inspecting the scattering amplitudes in Equation ( 12), we first observe that they are invariant under the following generalized gauge transformations of their numerators:

(14)Nj′=Nj+sjΔ,N~j′=N~j+sjΔ~.

We can determine the gauge parameters $\left(\Delta ,\tilde{\Delta }\right)$ by requiring the gauge-transformed numerators to obey the Jacobi identities $\sum_{j}N_{j}^{\prime }=0$ and $\sum_{j}{\tilde{N}}_{j}^{\prime }=0$ . Thus, we derive the following general solutions:

(15)Δ=−14Mn2∑jNj,Δ~=−14Mn2∑jN~j,

which realize the BCJ-respecting numerators $\left(N_{j}^{\prime },{\tilde{N}}_{j}^{\prime }\right)$ . Making high energy expansions on both sides of Equation ( 15), we derive the expressions of the gauge parameters $\left(\Delta ,\tilde{\Delta }\right)=\left(\Delta_{0}+\Delta_{1},{\tilde{\Delta }}_{0}+{\tilde{\Delta }}_{1}\right)$ at the LO and NLO:

(16)Δ0=149+7c2θcθcsc2θ,Δ~0=1417−c2θcθcsc2θ,Δ1=−231+c4θcθcsc4θMn2/s,Δ~1=−87+c2θcθcsc4θMn2/s.

With these, we further compute the new numerators $\left(N_{j}^{\prime },{\tilde{N}}_{j}^{\prime }\right)$ and derive explicitly the LO results in Equation ( 19) and the NLO results in [ 30].

For the 5d KK YM (YM5) and 5d KK GR (GR5) theories, we expect the double-copy correspondence between the KK gauge fields and KK graviton fields:

(17)Anaµ⊗Anaν⟶hnµν,Ana5⊗Ana5⟶hn55,Anaµ⊗Ana5⟶hnµ5.

The physical spin-2 KK graviton field $h_{n}^{µ\nu }$ arises from two copies of spin-1 KK gauge fields. The KK Goldstone boson $A_{n}^{a5}$ of the YM5 has its double-copy counterparts $h_{n}^{55}\left(=\phi_{n}\right)$ and $h_{n}^{\mu 5}\left(=A_{n}^{\mu }\right)$ which correspond to the scalar and vector KK Goldstone bosons in the compactified GR5. The double-copy correspondence between the longitudinal KK modes, $A_{L}^{an}⊗A_{L}^{an}⟶h_{L}^{n}$ , is *highly nontrivial even at the LO* of high energy expansion, because $\left(A_{L}^{an},h_{L}^{n}\right)$ do not exist in $M_{n}⟶0$ limit and the KK Goldstone bosons $\left(A_{5}^{an},\phi_{n}\right)$ become physical states in massless limit. Thus, this double-copy is consistently realized because we can use the KK GRET (GAET) to connect $h_{L}^{n}\left(A_{L}^{an}\right)$ amplitudes to the $\phi_{n}\left(A_{5}^{an}\right)$ amplitudes under $M_{n}/E⟶0$ limit.

Then, we extend the conventional double-copy method [ 25– 27] to the massive KK YM theory *under high energy expansion.* We apply the color-kinematics duality $C_{j}⟶N_{j}^{\prime }$ and $C_{j}⟶{\tilde{N}}_{j}^{\prime }$ to Equation ( 12) and construct the four-particle KK graviton (Goldstone) amplitudes:

(18a)M=∑jc0g2Nj0′+δNj′2sj=M0+δM,(18b)M~=∑jc0g2N~j0′+δN~j′2sj=M~0+δM~,

where we denote the scattering amplitudes $M\equiv M\left[h_{L}^{n}h_{L}^{n}⟶h_{L}^{n}h_{L}^{n}\right],\tilde{M}\equiv \tilde{M}\left[\phi_{n}\phi_{n}⟶\phi_{n}\phi_{n}\right]$ and $c_{0}$ is a conversion constant..

From Table 1 and using Equations ( 14) and ( 16), we find that the LO numerators $\left(N_{j}^{0}\prime ,{\tilde{N}}_{j}^{0}\prime \right)$ are *mass independent* and *equal to each other*:

(19a)Ns0′=N~s0′=s7+c2θcθ2sin2θ,(19b)Nt0′=N~t0′=−s42−15cθ+6c2θ−c3θ161−cθ,(19c)Nu0′=N~u0′=s42+15cθ+6c2θ+c3θ161+cθ.

This demonstrates the equivalence between the two leading-order KK amplitudes at $O\left(E^{0}M_{n}^{0}\right),T_{0}={\tilde{T}}_{0}$ , which explicitly realizes the KK GAET. With these and using our LO double-copy formulas in Equation ( 18), we can reconstruct the KK GRET:

(20)M0DC=M~0DC,

which is of $O\left(E^{2}M_{n}^{0}\right)$ . We stress that as expected, these LO amplitudes are *mass-independent* and thus the LO double-copy can universally hold. We further find that after setting the overall conversion constant of Equation ( 18) as $c_{0}=-\kappa^{2}/\left(24g^{2}\right)$ , the reconstructed LO KK amplitude $M_{0}\left({\tilde{M}}_{0}\right)$ just equals the LO KK Goldstone amplitude ( 8) and the corresponding LO longitudinal KK graviton amplitude [ 30]. Hence, our double-copy prediction ( 20) can prove (reconstruct) the GRET $M_{0}\left({\tilde{M}}_{0}\right)$ from the GAET $T_{0}={\tilde{T}}_{0}$ . We derived this GRET in Equation ( 9) by direct Feynman diagram calculations. Note that the KK GAET relation $T_{0}={\tilde{T}}_{0}$ can hold for general N -point longitudinal KK gauge (Goldstone) amplitudes [ 9, 20]. Hence, making double-copy on both sides of $T_{0}={\tilde{T}}_{0}$ can establish the GRET ( 20) to hold for N -point longitudinal KK graviton (Goldstone) amplitudes. From this, we can further establish *a new correspondence* between the two types of energy cancellations in the N -longitudinal KK gauge boson amplitudes and in the corresponding N -longitudinal KK graviton amplitudes (cf. the discussion around the end of Section 3).

Next, we use the double-copy formulas ( 18a) and ( 18b) to reconstruct the 4-point longitudinal KK graviton amplitude and the corresponding KK Goldstone boson amplitude at the NLO:

(21a)δMDCκ2Mn2=−51642+297c2θ+102c4θ+7c6θ768sin4θ,(21b)δM~DCκ2Mn2=−6386+3837c2θ+30c4θ−13c6θ768sin4θ.

They have *the same size* of $O\left(\kappa^{2}M_{n}^{2}\right)$ and *the same angular structure* of $\left(1,c_{2\theta },c_{4\theta },c_{6\theta }\right)\times \csc^{4}\theta$ as the original NLO amplitudes $\left(\delta M,\delta \tilde{M}\right)$ derived from Feynman diagram calculations [ 30], though their numerical coefficients still differ. Then, using Equation ( 21), we compute the difference between the two double-copied NLO amplitudes $\Delta M\left(\text{DC}\right)=\delta M-\delta \tilde{M}$ and compare it with the NLO amplitude difference $\Delta M\left(\text{GR}5\right)$ by Feynman diagram calculations in the KK GR5 theory:

(22a)ΔMGR5=−32κ2Mn219.5+c2θ,(22b)ΔMDC=−κ2Mn27+c2θ.

We find that they also have *the same size* of $O\left(\kappa^{2}M_{n}^{2}\right)$ and *the same angular structure* of $\left(1,c_{2\theta }\right)$ . Equation ( 22a) shows that the difference $\Delta M\left(\text{GR}5\right)$ between the original NLO amplitudes exhibits a striking precise cancellations of the angular structure $\left(1,c_{2\theta },c_{4\theta },c_{6\theta }\right)\times \csc^{4}\theta$ to $\left(1,c_{2\theta }\right)$ . Impressively, *our double-copied NLO amplitude difference* $\Delta M\left(DC\right)$ *in* Equation ( 22b) *can also realize the same type of the precise angular cancellations.*

The above extended NLO double-copy results ( 21) and ( 22b) are truly encouraging, because they already give *the correct structure* of the NLO KK amplitudes including *the precise cancellations* of the angular dependence in Equations ( 21) and ( 22). These strongly suggest that our massive KK double-copy approach is on the right track. Its importance is twofold: (i). In practice, for our proposed KK double-copy method *under high energy expansion*, the LO double-copy construction is the most important part because it newly establishes the GRET relation $M_{0}={\tilde{M}}_{0}$ (Equation ( 20)) from the GAET relation $T_{0}={\tilde{T}}_{0}$ (Equation ( 19) and below), as will be shown in Equation ( 34). The NLO KK graviton amplitudes are relevant only when we *estimate the size* of the residual term $M_{\Delta }$ of our GRET ( 7), and here, we do not need the precise form of $M_{\Delta }$ *except to justify its size* $M_{\Delta }=O\left(E^{0}M_{n}^{2}\right)$ by the double-copy construction (cf. Equation ( 33)). This proves that *the residual term* $M_{\Delta }$ *does belong to the NLO amplitudes and is negligible for our GRET formulation in the high energy limit.* Hence, we do not need any precise NLO double-copy here. (ii). In general, our current KK double-copy approach as the first serious attempt to construct the massive KK graviton amplitudes has given strong motivation and important guideline for a full resolution of the exact double-copy beyond the LO. Our further study has found out the reasons for the minor mismatch between the numerical coefficients of the double-copied NLO amplitudes ( 21) and that of the direct Feynman diagram calculations. One reason is due to the double-pole structure in the KK amplitudes (including exchanges of both the zero mode and KK modes) beyond the conventional massless theories, so the additional KK mass poles contribute to our mass-dependent NLO amplitudes and cause a mismatch. Another reason is because the exact polarization tensor of the (helicity-zero) longitudinal KK graviton is given by $\varepsilon_{L}^{\mu \nu }=\left(\epsilon_{+}^{\mu }\epsilon_{-}^{\nu }+\epsilon_{-}^{\mu }\epsilon_{+}^{\nu }+2\epsilon_{L}^{\mu }\epsilon_{L}^{\nu }\right)/\sqrt{6}$ [ 30], which contains not only the longitudinal product $\epsilon_{L}^{\mu }\epsilon_{L}^{\nu }$ but also the transverse products $\epsilon_{+}^{\mu }\epsilon_{-}^{\nu }+\epsilon_{-}^{\mu }\epsilon_{+}^{\nu }$ . So, other scattering amplitudes containing possible transversely polarized external KK gauge boson states should be included for a full double-copy besides the four-longitudinal KK gauge boson amplitude in Equation ( 12).

With these in minds, we have further used a first principle approach of the KK string theory in our recent study [ 39] to derive the extended massive KLT-like relations between the product of the KK open string amplitudes and the KK closed string amplitude. In the field theory limit, we can derive the exact double-copy relations between the product of the KK gauge boson amplitudes and the KK graviton amplitude at tree level [ 39]. In such exact double-copy relations, all the relevant helicity indices of the external KK gauge boson states are summed over to match the corresponding polarization tensors of the external KK graviton states. The double-pole structure is also avoided by first making the 5d compactification under $S^{1}$ (without orbifold) where the KK numbers $\left(\pm n=\pm 1,\pm 2,\pm 3,\cdots \right)$ are strictly conserved and the amplitudes are ensured to have single-pole structure. Then, we can define the ${\mathbb{Z}}_{2}$ -even (odd) KK states as $|n_{\pm }\rangle =\left(|+n\rangle \pm |-n\rangle \right)/\sqrt{2}$ and derive the amplitudes under $S^{1}/{\mathbb{Z}}_{2}$ compactification from the combinations of those amplitudes under the $S^{1}$ compactification [ 39]. Using this improved massive double-copy approach, we can exactly reconstruct all the massive KK graviton amplitudes at tree level. For the four longitudinal KK graviton amplitudes under $S^{1}/{\mathbb{Z}}_{2}$ , we derive the following (BCJ-type) exact massive double-copy formula:

(23)M=−κ264∑P∑j∑λk,λk′∏kCλkλk′NjPλkNjPλk′Dj,

where $\left\{N_{j}\right\}$ denote the kinematic numerators under the $S^{1}$ compactification and each external KK gauge boson has 3 helicity states (with $\lambda_{k}=\pm 1,L$ and k=1,2,3,4 ). We use $\text{P}=\left\{n_{1},n_{2},n_{3},n_{4}\right\}$ to label each possible combination of the KK numbers for external gauge bosons, which obey the condition of KK number conservation $\sum_{k=1}^{4}n_{k}=0$ . For the elastic KK scattering, we have

(24)P=±n,±n,∓n,∓n,±n,∓n,±n,∓n,±n,∓n,∓n,±n.

In Equation ( 23), $C_{\lambda_{k}\lambda_{k}^{\prime }}$ denotes the coefficients in the longitudinal polarization tensor of k th external KK graviton [ 30], $\varepsilon_{L,k}^{\mu \nu }=\sum C_{\lambda_{k}\lambda_{k}^{\prime }}\epsilon_{\lambda_{k}}^{\mu }\epsilon_{\lambda_{k}^{\prime }}^{\nu }$ , where $\lambda_{k},\lambda_{k}^{\prime }=\pm 1,L$ are the helicity indices for the k th external gauge boson. The denominator of ( 23) is defined as $D_{j}=s_{j}-M_{j}^{2}$ , where $s_{j}\in \left\{s,t,u\right\}$ and $M_{j}^{2}\in \left\{M_{n_{1}+n_{2}}^{2},M_{n_{1}+n_{4}}^{2},M_{n_{1}+n_{3}}^{2}\right\}$ .

Then, we make high energy expansion for the corresponding elastic amplitude of KK gauge bosons (under $S^{1}$ compactification) at the LO and NLO:

(25a)T=g2∑jCjNjPDj=g2∑jCjNj0,P+δNjPsj=T0+δT,(25b)NjP=DjsjNj0,P+δNjP.

With this, we expand the exact double-copy formula of the longitudinal KK graviton amplitude ( 23) under the high energy expansion of 1/s :

(26)M=−κ264∑P∑j∑λk,λk′∏kCλkλk′Djsj2×Nj0,Pλk+δNjPλkNj0,Pλk′+δNjPλk′=M0+δM.

It can be proven that the above double-copied LO amplitude $M_{0}$ is equivalent to the LO amplitude given in Equation ( 18a) [ 39]. We explicitly compute the above LO amplitude $M_{0}$ and find that $M_{0}$ just equals that of Equation ( 8) as well as Equation ( S22a) [ 30]. Then, we further compute the above double-copied NLO amplitude δM as follows:

(27)δM=−κ2Mn22561810+93c2θ+126c4θ+19c6θcsc4θ.

We find that this fully agrees with the exact NLO elastic KK graviton amplitude derived from the direct Feynman diagram calculation in Equation ( S22b) of the supplemental material [ 30]. The above analysis is an explicit demonstration that we can realize the exact (BCJ-type) massive double-copy construction of the four-point KK graviton amplitudes in Equation ( 23), as well as the precise double-copy of the KK graviton amplitudes ( 26) and ( 27) at both the LO and NLO of the high energy expansion. We will systematically pursue this new direction in our future work.

Finally, it is very encouraging that our improved massive double-copy construction of the longitudinal KK graviton (KK Goldstone) amplitude in Equation ( 18) is based on the pure longitudinal KK gauge (KK Goldstone) amplitude ( 12) alone, which can already give not only the precise LO KK graviton (KK Goldstone) amplitude, but also the *correct structure* of the NLO KK graviton (KK Goldstone) amplitude ( 21). In the following, we will propose another improved double-copy method to further reproduce the exact longitudinal KK graviton (KK Goldstone) amplitudes at the NLO and beyond. It only uses the amplitudes of pure longitudinal KK gauge bosons (KK Goldstone bosons) alone, hence it is practically simple and valuable. For this, we construct the following improved NLO numerators:

(28a)δNs″,δNt″,δNu″=δNs′,δNt′−z,δNu′+z,(28b)δN~s″,δN~t″,δN~u″=δN~s′,δN~t′−z~,δN~u′+z~,

where $\left(z,\tilde{z}\right)$ are functions of θ and can be determined by matching our improved NLO KK amplitudes of double-copy with the original NLO KK graviton (Goldstone) amplitudes of the GR5. Then, we solve $\left(z,\tilde{z}\right)$ as

(29a)z=Mn21390+603c2θ+66c4θ−11c6θ1213−12c2θ−c4θ,(29b)z~=Mn24546−3585c2θ+1086c4θ+c6θ1213−12c2θ−c4θ.

Note that the modified numerators ( 28) continue to hold the Jacobi identity. Because the corresponding NLO gauge (Goldstone) amplitudes $\left(\delta T^{\prime \prime },\delta {\tilde{T}}^{\prime \prime }\right)$ are modified only by terms of NLO, so we can still hold the general GAET identity $T^{\prime \prime }={\tilde{T}}^{\prime \prime }+T_{v}^{\prime \prime }$ by redefining the residual term as $T_{v}^{\prime \prime }=T_{v}-g^{2}\left(C_{t}/t-C_{u}/u\right)\left(z-\tilde{z}\right)$ . Using Equations ( 28)-( 29), we can reproduce the exact NLO KK gravitational scattering amplitudes [shown in Equations (S22a)-(S22b) of the Supplemental Material [ 30]]. This double-copy procedure can be further applied to higher orders beyond the NLO when needed.

## 6. GRET Residual Terms and Energy Cancellation

According to Table 1 and the generalized gauge transformation ( 14), we can explicitly deduce the equivalence between the KK gauge boson amplitude and the corresponding KK Goldstone boson amplitude,

(30)T0=T~0,

which belongs to the LO of $O\left(E^{0}M_{n}^{0}\right)$ . Using our double-copy method, we further derived the GRET relation $M_{0}={\tilde{M}}_{0}$ at the $O\left(E^{2}M_{n}^{0}\right)$ as shown in Equation ( 20). Thus, the residual terms of the GAET and the GRET ( 7) are given by the differences between the KK longitudinal amplitude and KK Goldstone amplitude at the NLO:

(31a)Tv≡∑TA5n,vn=δT−δT~=OMn2E2,(31b)MΔ≡∑MΔ~n,ϕn=δM−δM~=OE0Mn2.

The size of $T_{v}=O\left(M_{n}^{2}/E^{2}\right)$ can be easily understood by using our generalized power counting rule [ 30]. But, making the direct power counting gives $M_{\Delta }=O\left(E^{2}\right)$ for its individual amplitudes, which has the same energy dependence as the LO KK Goldstone amplitude ( 8).

We can further determine the size of the residual term $M_{\Delta }$ by the double-copy construction ( 18) based upon the KK gauge (Goldstone) boson scattering amplitudes of the YM5 theory alone (which are well understood [ 9, 19, 20, 40]). From Equation ( 18) and Table 1, we can estimate the residual term by power counting,

(32)MΔ=OδM,δM~=ONj0′δNj′sj,N~j0′δN~j′sj=OE0Mn2.

Thus, we deduce the double-copy correspondence between the residual term $T_{v}$ of the GAET and the residual term $M_{\Delta }$ of the GRET:

(33)Tv⟶MΔDC=OE0Mn2.

Hence, our double-copy construction proves that the GRET residual term $M_{\Delta }$ should have an energy cancellation $O\left(E^{2}\right)⟶O\left(E^{0}\right)$ among its individual amplitudes in Eq. ( 7b). This means that $M_{\Delta }$ is much smaller than the leading KK Goldstone amplitude ${\tilde{M}}_{0}=O\left(E^{2}M_{n}^{0}\right)$ .

From the above double-copy construction, we can establish a *new correspondence* from the GAET of the KK YM5 theory to the GRET of the 5d KK GR (GR5) theory:

(34)GAETYM5⟶GRETGR5.

We will present a systematically expanded analysis in the companion long paper [ 31], which includes our elaborations of the current key points and our extension of KLT relations [ 28] (along with CHY [ 41– 43]) to the double-copy construction of massive KK graviton amplitudes.

## 7. Conclusions

In this work, we newly formulated the geometric “Higgs” mechanism for the mass generation of Kaluza-Klein (KK) gravitons of the compactified 5d GR (GR5) theory at both the Lagrangian level and the scattering *S*-matrix level. Using a general $R_{\xi }$ gauge fixing of quantization, we proved that the KK graviton propagator is *free from* the longstanding problem of the vDVZ discontinuity [ 15, 16] in the conventional Fierz-Pauli massive gravity [ 17, 18] and demonstrated that the KK gravity theory *consistently realizes the mass-generation for spin-2 KK gravitons.*

We newly proposed and proved a gravitational equivalence theorem (GRET) which connects the N -point scattering amplitudes of the longitudinal KK gravitons to that of the gravitational KK Goldstone bosons. We computed the four-point scattering amplitudes of KK Goldstone bosons in comparison with the longitudinal KK graviton amplitudes and explicitly proved the equivalence between the leading amplitudes of the longitudinal KK graviton scattering and the corresponding KK Goldstone boson scattering at $O\left(E^{2}M_{n}^{0}\right)$ .

We developed a generalized power counting method for massive KK gauge and gravity theories. Using the GRET and the new power counting rules, we established *a general energy cancellation mechanism* under which the leading energy dependence of N -particle longitudinal KK graviton amplitudes $\left(\propto E^{2\left(N+1+L\right)}\right)$ must cancel down to a much lower energy power $\left(\propto E^{2\left(1+L\right)}\right)$ by an energy factor of $E^{2N}$ , where L denotes the loop number of the relevant Feynman diagram. For the case of longitudinal KK graviton scattering amplitudes with N=4 and L=0 , this proves the energy cancellations of $E^{10}⟶E^{2}$ .

Extending the conventional massless double-copy method [ 25– 27] to the compactified massive KK YM and GR theories, we derived the Jacobi-respecting numerators and constructed the amplitudes of longitudinal KK gravitons (KK Goldstone bosons) under high energy expansion. Using the double-copy method, we established *a new correspondence between the two energy cancellations* in the four-point longitudinal KK amplitudes: $E^{4}⟶E^{0}$ in the 5d KK YM gauge theory and $E^{10}⟶E^{2}$ in the 5d KK GR theory, which is connected to the double-copy correspondence between the GAET and GRET as we derived in Eq. ( 34). Furthermore, we analyzed the structure of the residual term $M_{\Delta }$ in the GRET ( 7) and further uncovered a new energy cancellation mechanism of $E^{2}⟶E^{0}$ therein.

Finally, we stress that the geometric Higgs mechanism is a general consequence of the KK compactification of extra spatial dimensions and should be realized for other KK gravity theories with more than one extra dimensions or nonflat extra dimensions. We note that our identity ( 6) results from the underlying gravitational diffeomorphism invariance and thus should generally hold for any compactified 5d KK GR theory with proper gauge-fixing functions. Thus, we expect that the GRET should generally hold for other 5d KK GR theories and take similar form as the present Eq. ( 7) [ 34]. For instance, we find that the geometric Higgs mechanism and the large energy-cancellations of the longitudinal KK graviton amplitudes are also realized in the compactified warped 5d space of the Randall-Sundrum model [ 5] and our GRET will work in the similar way. Following the current work, it is encouraging to further study these interesting issues in our future work [ 34].

## Data Availability

All data needed for the current work are presented in this paper and the Supplementary Materials. Additional data related to this paper may be requested from the authors.

## Supplementary Materials

Supplementary Materials In the Supplementary Materials, we include the following contents which provide the relevant technical details for the analyses presented in the main text of this paper. I. Kinematics of KK scattering. II. Feynman rules for 5d KK GR theory. III. Power counting and energy cancellations for KK graviton amplitudes. IV. KK graviton and Goldstone scattering amplitudes.
