# Supplementary material for: Gravitational Equivalence Theorem and Double-Copy for Kaluza-Klein Graviton Scattering Amplitudes
Source: Research (Wash D C). 2022 Jul 14;2022:9860945. doi: 10.34133/2022/9860945 (PMC12237073; doi:10.34133/2022/9860945)
Supplement: Supplementary Materials — In the Supplementary Materials, we include the following contents which provide the relevant technical details for the analyses presented in the main text of this paper. I. Kinematics of KK scattering. II. Feynman rules for 5d KK GR theory. III. Power counting and energy cancellations for KK graviton amplitudes. IV. KK graviton and Goldstone scattering amplitudes. [file 9860945.f1.pdf]

Gravitational Equivalence Theorem and Double-Copy for Kaluza-Klein Graviton Scattering Amplitudes

— Supplemental Material —

Yan-Feng Hang [1] and Hong-Jian He [1,2,3]

[1] Tsung-Dao Lee Institute & School of Physics and Astronomy,
Key Laboratory for Particle Astrophysics and Cosmology (MOE),
Shanghai Key Laboratory for Particle Physics and Cosmology,
Shanghai Jiao Tong University, Shanghai, China

[2] Institute of Modern Physics & Physics Department, Tsinghua University, Beijing, China

[3] Center for High Energy Physics, Peking University, Beijing, China

(yfhang@sjtu.edu.cn, hjhe@sjtu.edu.cn)

This Supplemental Material provides in detail the relevant formulas and Feynman rules for the analyses of the KK scattering amplitudes in the compactified 5d Yang-Mills (YM5) theory and the compactified 5d General Relativity (GR5) theory.

Contents

1. Kinematics of KK Scattering 1

2. Feynman Rules for 5d KK GR Theory 2

3. Power Counting and Energy Cancellations for KK Graviton Scattering Amplitudes 4

4. KK Graviton and Goldstone Scattering Amplitudes 6

4.1. KK Graviton and Goldstone Amplitudes from Feynman Diagrams 6

4.2. KK Graviton and Goldstone Amplitudes from Extended Double-Copy 7

1 Kinematics of KK Scattering

We consider $2\to 2$ KK scattering process, with the four-momentum of each external state obeying the on-shell condition $p_j^2 = -M_j^2$, $(j = 1, 2, 3, 4)$. We number the external lines clockwise, with their momenta being out-going. Thus, the energy-momentum conservation gives $\sum_j p_j = 0$, and the physical momenta of the two incident particles equal $-p_1$ and $-p_2$, respectively. For illustration, we take the elastic scattering $X_n X_n \to X_n X_n$ $(n \geqslant 0)$ as an example, where X_n denotes any given KK state of level-n and has $M_j = M_n$. For the KK theory, the external particle has mass M_n for a given KK-state of level-n. Thus, in the center-of-mass frame, we define the momenta as follows:

$$
\begin{aligned}
p_1^\mu &= -E\,(1,0,0,\beta)\ , \qquad & p_2^\mu &= -E\,(1,0,0,-\beta)\ , \\
p_3^\mu &= E\,(1,\beta s_\theta,0,\beta c_\theta)\ , \qquad & p_4^\mu &= E\,(1,-\beta s_\theta,0,-\beta c_\theta)\ ,
\end{aligned}
\tag{S1}
$$

where $(s_\theta,\, c_\theta)=(\sin\theta,\, \cos\theta)$ and $\beta=(1-M_n^2/E^2)^{1/2}$. With the above, we can define the following three Mandelstam variables:

$$s=-(p_1+p_2)^2=4E^2, \quad t=-(p_1+p_4)^2=-\tfrac{1}{2}s\beta^2(1+c_\theta), \quad u=-(p_1+p_3)^2=-\tfrac{1}{2}s\beta^2(1-c_\theta). \quad \text{(S2)}$$

Then, using the on-shell condition $E^2=E^2\beta^2+M_n^2$, we define a new set of mass-independent Mandelstam variables as follows:

$$s_0=4E^2\beta^2, \qquad t_0=-\frac{s_0}{2}(1+c_\theta), \qquad u_0=-\frac{s_0}{2}(1-c_\theta), \quad \text{(S3)}$$

where $s_0=s-4M_n^2$, and thus $(s_0,\, t_0,\, u_0)=(s\beta^2,\, t,\, u)$. Summing up the Mandelstam variables (S2) and (S3) gives the following relations:

$$s+t+u=4M_n^2\,, \qquad s_0+t_0+u_0=0\,. \quad \text{(S4)}$$

As we mentioned in the text, a massive KK graviton has 5 helicity states ($\lambda=\pm2,\pm1,0$). Their polarization tensors take the following forms:

$$\varepsilon_{\pm2}^{\mu\nu}=\epsilon_\pm^\mu\epsilon_\pm^\nu\,, \qquad \varepsilon_{\pm1}^{\mu\nu}=\frac{1}{\sqrt{2}}\left(\epsilon_\pm^\mu\epsilon_L^\nu+\epsilon_L^\mu\epsilon_\pm^\nu\right), \qquad \varepsilon_L^{\mu\nu}=\frac{1}{\sqrt{6}}\left(\epsilon_+^\mu\epsilon_-^\nu+\epsilon_-^\mu\epsilon_+^\nu+2\epsilon_L^\mu\epsilon_L^\nu\right), \quad \text{(S5)}$$

where $(\epsilon_\pm^\mu,\, \epsilon_L^\mu)$ are the (transverse, longitudinal) polarization vectors of a vector boson with the same 4-momentum p^μ. These polarization tensors obey the traceless and orthonormal conditions. They are also orthogonal to the KK graviton's 4-momentum p^μ. Hence, the following conditions are realized:

$$\eta_{\mu\nu}\varepsilon^{\mu\nu}=0\,, \qquad \varepsilon_\lambda^{\mu\nu}\varepsilon_{\lambda',\mu\nu}^{*}=\delta_{\lambda\lambda'}\,, \qquad p_\mu\varepsilon^{\mu\nu}=0\,, \quad \text{(S6)}$$

where the helicity indices of each KK graviton are $\lambda,\lambda'=\pm2,\pm1,0$.

2 Feynman Rules for 5d KK GR Theory

In this section, we summarize the relevant Feynman rules [1] including propagators and vertices which are used for the amplitude calculations in the text of this Letter.

We first give the propagators in R_ξ gauge for KK graviton ($h_n^{\mu\nu}$) and KK Goldstone bosons ($\mathcal{A}_n^\mu$, ϕ_n) as follows:

$$\begin{aligned}\mathcal{D}_{nm}^{\mu\nu\alpha\beta}(p) =& -\frac{\mathrm{i}\delta_{nm}}{2}\left\{\frac{\eta^{\mu\alpha}\eta^{\nu\beta}+\eta^{\mu\beta}\eta^{\nu\alpha}-\eta^{\mu\nu}\eta^{\alpha\beta}}{p^2+M_n^2}+\frac{1}{3}\left[\frac{1}{p^2+M_n^2}-\frac{1}{p^2+(3\xi_n-2)M_n^2}\right]\left(\eta^{\mu\nu}-\frac{2p^\mu p^\nu}{M_n^2}\right)\left(\eta^{\alpha\beta}-\frac{2p^\alpha p^\beta}{M_n^2}\right)\right.\\ &+\frac{1}{M_n^2}\left(\frac{1}{p^2+M_n^2}-\frac{1}{p^2+\xi_n M_n^2}\right)\left(\eta^{\mu\alpha}p^\nu p^\beta+\eta^{\mu\beta}p^\nu p^\alpha+\eta^{\nu\alpha}p^\mu p^\beta+\eta^{\nu\beta}p^\mu p^\alpha\right)\\ &\left.+\frac{4p^\mu p^\nu p^\alpha p^\beta}{\xi_n M_n^4}\left(\frac{1}{p^2+\xi_n^2M_n^2}-\frac{1}{p^2+\xi_n M_n^2}\right)\right\}, \quad \text{(S7a)}\end{aligned}$$

$$\mathcal{D}_{nm}^{\mu\nu}(p)=\frac{-\mathrm{i}\delta_{nm}}{p^2+\xi_n M_n^2}\left[\eta^{\mu\nu}-\frac{p^\mu p^\nu(1-\xi_n)}{p^2+\xi_n^2M_n^2}\right], \qquad \mathcal{D}_{nm}(p)=\frac{-\mathrm{i}\delta_{nm}}{p^2+(3\xi_n-2)M_n^2}\,. \quad \text{(S7b)}$$

For the Feynman-'t Hooft gauge ($\xi_n=1$), the above propagators reduce to the simple forms [cf. Eq.(5) in the main text].

Next, we make the following Fourier expansions for the 5d graviton fields in terms of their zero modes and KK states:

$$\hat{h}^{\mu\nu}(x^\rho,x^5)=\frac{1}{\sqrt{L}}\left[h_0^{\mu\nu}(x^\rho)+\sqrt{2}\sum_{n=1}^{\infty}h_n^{\mu\nu}(x^\rho)\cos\frac{n\pi x^5}{L}\right], \quad \text{(S8a)}$$

$$\hat{h}^{\mu 5}(x^\rho, x^5) \;=\; \sqrt{\frac{2}{L}}\sum_{n=1}^{\infty} h_n^{\mu 5}(x^\rho)\sin\frac{n\pi x^5}{L}\,, \tag{S8b}$$

$$\hat{\phi}(x^\rho, x^5) \;=\; \frac{1}{\sqrt{L}}\left[\phi_0(x^\rho) + \sqrt{2}\sum_{n=1}^{\infty}\phi_n(x^\rho)\cos\frac{n\pi x^5}{L}\right]. \tag{S8c}$$

With these, we list the relevant 4d effective Lagrangians including both cubic and quartic interactions which are used for our analyses:

$$\begin{aligned}
\mathcal{L}_1[h\phi^2] = \frac{\kappa}{\sqrt{2}}\sum_{n,m,\ell=1}^{\infty}\Big\{& a_1\big[\sqrt{2}(h_0^{\mu\nu}\partial_\mu\phi_0\partial_\nu\phi_0 + h_0^{\mu\nu}\partial_\mu\phi_m\partial_\nu\phi_\ell\delta_{m\ell} + h_n^{\mu\nu}\partial_\mu\phi_m\partial_\nu\phi_0\delta_{nm} + h_n^{\mu\nu}\partial_\mu\phi_0\partial_\nu\phi_\ell\delta_{n\ell}) \\
&+ h_n^{\mu\nu}\partial_\mu\phi_m\partial_\nu\phi_\ell\,\Delta_3(n,m,\ell)\big] + a_2\big[\sqrt{2}(h_0^{\mu\nu}\phi_0\partial_\mu\partial_\nu\phi_0 + h_0^{\mu\nu}\phi_m\partial_\mu\partial_\nu\phi_\ell\delta_{m\ell} + h_n^{\mu\nu}\phi_m\partial_\mu\partial_\nu\phi_0\delta_{nm} \\
&+ h_n^{\mu\nu}\phi_0\partial_\mu\partial_\nu\phi_\ell\delta_{n\ell}) + h_n^{\mu\nu}\phi_m\partial_\mu\partial_\nu\phi_\ell\Delta_3(n,m,\ell)\big] + a_3\big[\sqrt{2}(h_0\partial_\mu\phi_0\partial^\mu\phi_0 + h_0\partial_\mu\phi_m\partial^\mu\phi_\ell\delta_{m\ell} \\
&+ h_n\partial_\mu\phi_m\partial^\mu\phi_0\delta_{nm} + h_n\partial_\mu\phi_0\partial^\mu\phi_\ell\delta_{n\ell}) + h_n\partial_\mu\phi_m\partial^\mu\phi_\ell\,\Delta_3(n,m,\ell)\big] + a_4\big[\sqrt{2}(h_0\phi_0\partial_\mu^2\phi_0 \\
&+ h_0\phi_m\partial_\mu^2\phi_\ell\delta_{m\ell} + h_n\phi_m\partial_\mu^2\phi_0\delta_{nm} + h_n\phi_0\partial_\mu^2\phi_\ell\delta_{n\ell}) + h_n\phi_m\partial_\mu^2\phi_\ell\,\Delta_3(n,m,\ell)\big] \\
&+ a_5 M_m M_\ell\big[\sqrt{2}h_0\phi_m\phi_\ell\delta_{m\ell} + h_n\phi_m\phi_\ell\,\widetilde{\Delta}_3(n,m,\ell)\big] - a_6 M_\ell^2\big[\sqrt{2}(h_0\phi_m\phi_\ell\delta_{m\ell} + h_n\phi_0\phi_\ell\delta_{n\ell}) \\
&+ h_n\phi_m\phi_\ell\,\Delta_3(n,m,\ell)\big]\Big\}\,,
\end{aligned} \tag{S9a}$$

$$\begin{aligned}
\mathcal{L}_1[\mathcal{A}\phi^2] = -\frac{\kappa}{\sqrt{2}}\sum_{n,m,\ell=1}^{\infty}\Big\{& b_1 M_\ell\big[\sqrt{2}\mathcal{A}_n^\mu\partial_\mu\phi_0\phi_\ell\delta_{n\ell} + \mathcal{A}_n^\mu\partial_\mu\phi_m\phi_\ell\,\widetilde{\Delta}_3'(n,m,\ell)\big] + b_2 M_\ell\big[\sqrt{2}\mathcal{A}_n^\mu\phi_0\partial_\mu\phi_\ell\delta_{n\ell} \\
&+ \mathcal{A}_n^\mu\phi_m\partial_\mu\phi_\ell\,\widetilde{\Delta}_3'(n,m,\ell)\big]\Big\}\,,
\end{aligned} \tag{S9b}$$

$$\begin{aligned}
\mathcal{L}_1[\phi^3] = \frac{\kappa}{\sqrt{2}}\sum_{n,m,\ell=1}^{\infty}\Big\{& c_1\big[\sqrt{2}(\phi_0(\partial_\mu\phi_0)^2 + \phi_0\partial_\mu\phi_m\partial^\mu\phi_\ell\,\delta_{m\ell} + \phi_n\partial_\mu\phi_0\partial^\mu\phi_m\delta_{nm} + \phi_n\partial_\mu\phi_0\partial^\mu\phi_\ell\,\delta_{n\ell}) \\
&+ \phi_n\partial_\mu\phi_m\partial^\mu\phi_\ell\,\Delta_3(n,m,\ell)\big] + c_2 M_m M_\ell\big[\sqrt{2}\,\phi_0\phi_m\phi_\ell\delta_{m\ell} + \phi_n\phi_m\phi_\ell\widetilde{\Delta}_3(n,m,\ell)\big]\Big\}\,,
\end{aligned} \tag{S9c}$$

$$\begin{aligned}
\mathcal{L}_2[\phi^4] = \frac{\kappa^2}{2}\sum_{n,m,\ell,k=1}^{\infty}\Big\{& d_1\Big\{2(\phi_0\partial_\mu\phi_0)^2 + 2\big[(\partial_\mu\phi_0)^2\,\phi_n\phi_m\delta_{nm} + \phi_0\partial_\mu\phi_0\phi_n\partial^\mu\phi_\ell\delta_{n\ell} + \phi_0\partial_\mu\phi_0\phi_n\partial^\mu\phi_k\delta_{nk} \\
&+ \phi_0\partial_\mu\phi_0\phi_m\partial^\mu\phi_k\delta_{mk} + \phi_0\partial_\mu\phi_0\phi_m\partial^\mu\phi_\ell\delta_{m\ell} + (\phi_0)^2\partial_\mu\phi_\ell\partial^\mu\phi_k\delta_{\ell k}\big] + \sqrt{2}\,\big[\,\partial_\mu\phi_0\phi_n\phi_m\partial^\mu\phi_\ell\Delta_3(n,m,\ell) \\
&+ \partial_\mu\phi_0\phi_n\phi_m\partial^\mu\phi_k\,\Delta_3(n,m,k) + \partial_\mu\phi_0\phi_n\phi_\ell\partial^\mu\phi_k\Delta_3(n,\ell,k) + \phi_0\phi_m\partial_\mu\phi_\ell\partial^\mu\phi_k\Delta_3(m,\ell,k)\,\big] \\
&+ \phi_n\phi_m\partial_\mu\phi_\ell\partial^\mu\phi_k\,\Delta_4(n,m,\ell,k)\Big\} + d_2 M_\ell M_k\big[2(\phi_0)^2\phi_\ell\phi_k\delta_{\ell k} + \sqrt{2}\phi_0\phi_m\phi_\ell\phi_k\widetilde{\Delta}_3(m,\ell,k) \\
&+ \sqrt{2}\phi_0\phi_n\phi_\ell\phi_k\,\widetilde{\Delta}_3(n,\ell,k) + \phi_n\phi_m\phi_\ell\phi_k\,\widetilde{\Delta}_4(n,m,\ell,k)\big]\Big\}\,,
\end{aligned} \tag{S9d}$$

where the delta functions $(\Delta_j, \widetilde{\Delta}_j)$ are defined as follows:

$$\begin{aligned}
\Delta_3(n,m,\ell) \;&=\; \delta(n{+}m{-}\ell) + \delta(n{-}m{-}\ell) + \delta(n{-}m{+}\ell)\,, \\
\widetilde{\Delta}_3(n,m,\ell) \;&=\; \delta(n{+}m{-}\ell) - \delta(n{-}m{-}\ell) + \delta(n{-}m{+}\ell)\,, \\
\widetilde{\Delta}_3'(n,m,\ell) \;&=\; \delta(n{+}m{-}\ell) - \delta(n{-}m{+}\ell) + \delta(n{-}m{-}\ell)\,, \\
\Delta_4(n,m,\ell,k) \;&=\; \delta(n{+}m{+}\ell{-}k) + \delta(n{+}m{-}\ell{-}k) + \delta(n{-}m{+}\ell{-}k) + \delta(n{-}m{-}\ell{-}k) \\
&\quad + \delta(n{-}m{-}\ell{+}k) + \delta(n{+}m{-}\ell{+}k) + \delta(n{-}m{+}\ell{+}k)\,, \\
\widetilde{\Delta}_4(n,m,\ell,k) \;&=\; \delta(n{+}m{+}\ell{-}k) - \delta(n{+}m{-}\ell{-}k) + \delta(n{-}m{+}\ell{-}k) - \delta(n{-}m{-}\ell{-}k) \\
&\quad + \delta(n{-}m{-}\ell{+}k) - \delta(n{+}m{-}\ell{+}k) + \delta(n{-}m{+}\ell{+}k)\,.
\end{aligned} \tag{S10}$$

Then, we derive the Feynman rules based on the interaction Lagrangians in the above Eq.(S9). We present the relevant 3-point and 4-point vertices as follows:

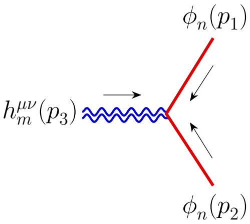

$$= \frac{-\mathrm{i}\kappa}{\sqrt{1+\delta_{2n,m}}}\begin{bmatrix} a_1(p_1^\mu p_2^\nu + p_1^\nu p_2^\mu) \\ +\ a_2(p_1^\mu p_1^\nu + p_2^\mu p_2^\nu) \\ +\ 2a_3\eta^{\mu\nu}(p_1\cdot p_2) \\ -\ 2\tilde{a}_4\eta^{\mu\nu}M_n^2 \end{bmatrix}, \tag{S11a}$$

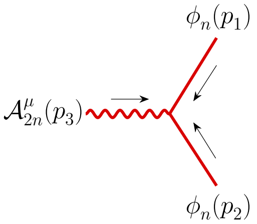

$$= -\frac{\kappa(b_1+b_2)M_n}{\sqrt{2}}(p_1^\mu + p_2^\mu)\,, \tag{S11b}$$

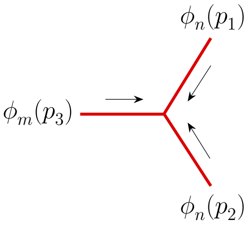

$$= \begin{cases} m=0:\ \mathrm{i}\,2\kappa\big[c_1(p_1^2+p_2^2+p_1\cdot p_2)+c_2M_n^2\big] \\ \qquad\xrightarrow{\text{on-shell}} \mathrm{i}\,2\kappa\big[c_1(p_1\cdot p_2)-(2c_1-c_2)M_n^2\big], \\ m=2n:\ -\mathrm{i}\sqrt{2}\,\kappa\big[c_1(p_1\cdot p_2)+c_2M_n^2\big], \end{cases} \tag{S11c}$$

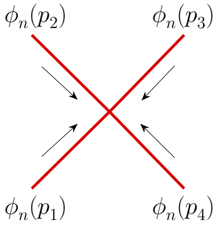

$$\begin{aligned} &= \mathrm{i}\,6\kappa^2\big[d_1(p_1^2+p_2^2+p_1\cdot p_2-p_3\cdot p_4)+2d_2M_n^2\big] \\ &\xrightarrow{\text{on-shell}} -\mathrm{i}\,12\kappa^2(d_1-d_2)M_n^2\,. \end{aligned} \tag{S11d}$$

where $\tilde{a}_4 = a_4 + (-1)^{\delta_{2n,m}}a_5 - a_6$ with $m = 0,\ 2n$ in Eq.(S11a).

3 Power Counting and Energy Cancellations for KK Graviton Scattering Amplitudes

We consider a S-matrix element $\mathbb{S}$ having $\mathcal{E}$ external states and L loops ($L \geqslant 0$). Extending Weinberg's power counting rule for the ungauged nonlinear σ-model of the low energy QCD [2], we develop generalized power counting approach [1] for the KK gravity theory. The mass dimension of a given scattering amplitude $\mathbb{S}$ in 4d is counted as

$$D_{\mathbb{S}} = 4 - \mathcal{E}\,, \tag{S12}$$

where the number of external states $\mathcal{E} = \mathcal{E}_B + \mathcal{E}_F$ with $\mathcal{E}_B\,(\mathcal{E}_F)$ representing the total number of external bosonic (fermionic) states. In addition, we only consider the SM fermions whose masses are much smaller than the scattering energy. We denote the number of vertices of type-j as $\mathcal{V}_j$. Each

vertex of type-j contains d_j derivatives, b_j bosonic lines and f_j fermionic lines. Then, the energy dependence of coupling constant in $\mathbb{S}$ is given by

$$D_C = \sum_j \mathcal{V}_j \big(4 - d_j - b_j - \tfrac{3}{2} f_j\big). \tag{S13}$$

For each Feynman diagram in the amplitude $\mathbb{S}$, we denote the number of the internal lines as $I = I_B + I_F$ with I_B (I_F) being the number of the internal bosonic (fermionic) lines. Thus, we have the following general relations:

$$L = 1 + I - \mathcal{V}\,, \quad \mathcal{V} = \sum_j \mathcal{V}_j\,, \quad \sum_j \mathcal{V}_j b_j = 2I_B + \mathcal{E}_B\,, \quad \sum_j \mathcal{V}_j f_j = 2I_F + \mathcal{E}_F\,, \tag{S14}$$

where $\mathcal{V}$ is the total number of vertices in a given Feynman diagram. The $\mathbb{S}$ may include $\mathcal{E}_{h_L}$ external longitudinal KK graviton states. Thus, taking Eqs.(S12)-(S14), we deduce the leading energy-power dependence as follows:

$$D_E = D_{\mathbb{S}} - D_C = 2\mathcal{E}_{h_L} + (2L+2) + \sum_j \mathcal{V}_j \big(d_j - 2 + \tfrac{1}{2} f_j\big). \tag{S15}$$

For the pure longitudinal KK graviton scattering amplitude with N external states, we have $\mathcal{E}_{h_L} = N$ and $f_j = 0$. Each pure KK graviton vertex always contains two partial derivatives and thus $d_j = 2$. For the loop level ($L \geqslant 1$), the amplitude may contain gravitational ghost loop which involves graviton-ghost-antighost vertex, but the number of partial derivatives d_j should be no more than two. While for the gravitational KK Goldstone boson scattering amplitude, its leading energy dependence is given by the diagrams containing the cubic vertices of type $h_n^{\mu\nu}$-ϕ_m-ϕ_ℓ and the pure graviton self-interaction vertices, where each of these vertices includes two derivatives ($d_j = 2$). Hence, we can derive the power counting formula (S15) as:

$$D_E[Nh_L^n] = 2(N+1) + 2L\,, \qquad D_E[N\phi_n] = 2 + 2L\,, \tag{S16}$$

where the notation $[Nh_L^n]$ and $[N\phi_n]$ denote the N external longitudinal KK graviton states and N external KK Goldstone states respectively.

Comparing the energy power counting formulas for KK graviton and KK Goldstone in Eq.(S16), we note that their difference arises from the leading energy-dependence of the polarization tensors $\varepsilon_L^{\mu\nu} \sim k^\mu k^\nu / M_n^2$ for the N external longitudinal KK gravitons in the high energy scattering:

$$D_E[Nh_L^n] - D_E[N\phi_n] = 2N\,. \tag{S17}$$

Finally, we examine the leading energy dependence of the individual amplitudes in the residual term $\mathcal{M}_\Delta$ of the GRET [cf. Eq.(7) in main text]. A typical leading amplitude can be $\mathcal{M}[\tilde{v}_{n_1}, \cdots, \tilde{v}_{n_N}]$, in which all the external states are KK gravitons contracted with $\tilde{v}^{\mu\nu} = \varepsilon_L^{\mu\nu} - \varepsilon_S^{\mu\nu} = \mathcal{O}(E^0)$, such as $\tilde{v}_n = \tilde{v}_{\mu\nu} h_n^{\mu\nu}$. Hence, the leading energy dependence of this amplitude yields:

$$D_E[N\tilde{v}_n] = 2 + 2L\,, \tag{S18}$$

which gives the same energy power dependence as $D_E[N\phi_n]$.

4 KK Graviton and Goldstone Scattering Amplitudes

In this section, we first present the four-point scattering amplitudes of KK gravitons (Goldstone bosons) at the LO and NLO of the high energy expansion, which are obtained from computing the Feynman diagrams. Then, we present the four-point scattering amplitudes of the KK gauge bosons (Goldstone bosons) at the LO and NLO under two kinds of high energy expansions. Using these we provide the detailed formulas for our improved massive double-copy construction of the KK graviton (Goldstone) amplitudes which are duscussed in the main text.

4.1 KK Graviton and Goldstone Amplitudes from Feynman Diagrams

In this subsection, we summarize the full elastic amplitudes of the four longitudinal KK graviton scattering [3] and of the four gravitational KK Goldstone boson scattering [1]. For the purpose of our double-copy analysis, we express these amplitudes in terms of the dimensionless variable $\bar{s}$:

$$\mathcal{M}[4h_L^n] = -\frac{\kappa^2 M_n^2 (X_0 + X_2 c_{2\theta} + X_4 c_{4\theta} + X_6 c_{6\theta}) \csc^2\theta}{512\,\bar{s}\,(\bar{s}-4)[\bar{s}^2 - (\bar{s}-4)^2 c_{2\theta} + 24\bar{s} + 16]}\,, \tag{S19a}$$

$$\widetilde{\mathcal{M}}[4\phi_n] = -\frac{\kappa^2 M_n^2 (\widetilde{X}_0 + \widetilde{X}_2 c_{2\theta} + \widetilde{X}_4 c_{4\theta} + \widetilde{X}_6 c_{6\theta}) \csc^2\theta}{512\,\bar{s}\,(\bar{s}-4)[\bar{s}^2 - (\bar{s}-4)^2 c_{2\theta} + 24\bar{s} + 16]}\,, \tag{S19b}$$

where $\bar{s} = s/M_n^2$ and $c_{n\theta} = \cos(n\theta)$. In the above, the coefficients $(X_j,\, \widetilde{X}_j)$ are defined as follows:

$$\begin{aligned}
X_0 &= -2(255\bar{s}^5 + 2824\bar{s}^4 - 19936\bar{s}^3 + 39936\bar{s}^2 - 256\bar{s} + 14336)\,,\\
X_2 &= 429\bar{s}^5 - 10152\bar{s}^4 + 30816\bar{s}^3 - 27136\bar{s}^2 - 49920\bar{s} + 34816\,,\\
X_4 &= 2(39\bar{s}^5 - 312\bar{s}^4 - 2784\bar{s}^3 - 11264\bar{s}^2 + 26368\bar{s} - 2048)\,,\\
X_6 &= 3\bar{s}^5 + 40\bar{s}^4 + 416\bar{s}^3 - 1536\bar{s}^2 - 3328\bar{s} - 2048\,,\\
\widetilde{X}_0 &= -2(255\bar{s}^5 + 8248\bar{s}^4 - 4144\bar{s}^3 + 79104\bar{s}^2 + 642560\bar{s} + 69632)\,,\\
\widetilde{X}_2 &= 429\bar{s}^5 + 4152\bar{s}^4 + 21216\bar{s}^3 - 150016\bar{s}^2 + 1142016\bar{s} + 182272\,,\\
\widetilde{X}_4 &= 2(39\bar{s}^5 - 1992\bar{s}^4 + 17808\bar{s}^3 - 58112\bar{s}^2 + 70144\bar{s} - 20480)\,,\\
\widetilde{X}_6 &= 3\bar{s}^5 - 56\bar{s}^4 + 416\bar{s}^3 - 1536\bar{s}^2 + 2816\bar{s} - 2048\,.
\end{aligned} \tag{S20}$$

Then, we expand the KK graviton and KK Goldstone scattering amplitudes (S19a)-(S19b) under the high energy expansion of $1/s$:

$$\mathcal{M}[4h_L^n] \;=\; \mathcal{M}_0[4h_L^n] + \delta\mathcal{M}[4h_L^n]\,, \tag{S21a}$$

$$\widetilde{\mathcal{M}}[4\phi_n] \;=\; \widetilde{\mathcal{M}}_0[4\phi_n] + \delta\widetilde{\mathcal{M}}[4\phi_n]\,, \tag{S21b}$$

where the LO and NLO KK amplitudes take the following forms,

$$\mathcal{M}_0[4h_L^n] \;=\; \widetilde{\mathcal{M}}_0[4\phi_n] = \frac{3\kappa^2}{128}\, s\,(7 + c_{2\theta})^2 \csc^2\theta\,, \tag{S22a}$$

$$\delta\mathcal{M}[4h_L^n] \;=\; -\frac{\kappa^2 M_n^2}{256}(1810 + 93c_{2\theta} + 126c_{4\theta} + 19c_{6\theta}) \csc^4\theta\,, \tag{S22b}$$

$$\delta\widetilde{\mathcal{M}}[4\phi_n] \,=\, -\frac{\kappa^2 M_n^2}{256}(-902+3669c_{2\theta}-714c_{4\theta}-5\,c_{6\theta})\csc^4\theta\,. \tag{S22c}$$

If we make instead the high energy expansion in terms of $1/s_0$, we derive the following LO and NLO KK amplitudes:

$$\mathcal{M}_0'[4h_L^n] \,=\, \widetilde{\mathcal{M}}_0'[4\phi_n] \,=\, \frac{3\kappa^2}{128}\,s_0\,(7+c_{2\theta})^2\csc^2\theta\,, \tag{S23a}$$

$$\delta\mathcal{M}'[4h_L^n] \,=\, -\frac{\kappa^2 M_n^2}{128}(650+261c_{2\theta}+102c_{4\theta}+11c_{6\theta})\csc^4\theta\,, \tag{S23b}$$

$$\delta\widetilde{\mathcal{M}}'[4\phi_n] \,=\, -\frac{\kappa^2 M_n^2}{128}(-706+2049c_{2\theta}-318c_{4\theta}-c_{6\theta})\csc^4\theta\,, \tag{S23c}$$

where $s_0 = s-4M_n^2$. We see that the $1/s_0$ expansion has shifted a hidden $\mathcal{O}(M_n^2)$ subleading term (contained in $s=s_0+4M_n^2$) from the LO amplitudes (S22a) into the NLO amplitudes (S23b)-(S23c). But this rearrangement in Eqs.(S23a)-(S23c) does not affect the difference between the two NLO amplitudes. Thus, we can deduce the contribution of the residual terms by computing the amplitude-difference from either Eqs.(S22b)-(S22c) or Eqs.(S23b)-(S23c) as follows:

$$\mathcal{M}_\Delta \,=\, \delta\mathcal{M}[4h_L^n]-\delta\widetilde{\mathcal{M}}[4\phi_n] \,=\, -\frac{3\kappa^2 M_n^2}{2}\left(\frac{39}{2}+c_{2\theta}\right). \tag{S24}$$

This provides Eq.(22a) in the main text.

4.2 KK Graviton and Goldstone Amplitudes from Extended Double-Copy

We expand the scattering amplitudes under the high energy expansion in terms of M_n^2/s. Thus, we can express 4-point elastic KK gauge boson (Goldstone) amplitudes as follows:

$$\mathcal{T}[4A_L^n] \,=\, g^2\left(\frac{\mathcal{C}_s\mathcal{N}_s}{s}+\frac{\mathcal{C}_t\mathcal{N}_t}{t}+\frac{\mathcal{C}_u\mathcal{N}_u}{u}\right), \tag{S25a}$$

$$\widetilde{\mathcal{T}}[4A_5^n] \,=\, g^2\left(\frac{\mathcal{C}_s\widetilde{\mathcal{N}}_s}{s}+\frac{\mathcal{C}_t\widetilde{\mathcal{N}}_t}{t}+\frac{\mathcal{C}_u\widetilde{\mathcal{N}}_u}{u}\right), \tag{S25b}$$

which are invariant under the following generalized gauge-transformations:

$$\mathcal{N}_j' = \mathcal{N}_j + s_j\,\Delta\,, \qquad \widetilde{\mathcal{N}}_j' = \widetilde{\mathcal{N}}_j + s_j\,\widetilde{\Delta}\,. \tag{S26}$$

The above Eqs.(S25)-(S26) are given in Eqs.(12)(14) of the main text. This allows us to find proper solutions of $\{\Delta\,,\widetilde{\Delta}\}$ which ensure the gauge-transformed NLO numerators ($\delta\mathcal{N}_j'$, $\delta\widetilde{\mathcal{N}}_j'$) to obey the kinematic Jacobi identity, as we demonstrated in Eqs.(15)-(16) of the main text (cf. Sec.V). Thus, from these we can derive the gauge-transformed NLO numerators for the elastic KK gauge boson amplitude:

$$\delta\mathcal{N}_s' = -\tfrac{1}{4}M_n^2(246\,c_\theta+7\,c_{3\theta}+3\,c_{5\theta})\csc^4\theta\,, \tag{S27a}$$

$$\delta\mathcal{N}_t' = \frac{M_n^2(131-8c_\theta-4c_{2\theta}+8c_{3\theta}+c_{4\theta})}{8(1-c_\theta)^2}\,, \tag{S27b}$$

$$\delta\mathcal{N}_u' = -\frac{M_n^2(131+8c_\theta-4c_{2\theta}-8c_{3\theta}+c_{4\theta})}{8(1+c_\theta)^2}\,, \tag{S27c}$$

and the gauge-transformed NLO numerators for the corresponding KK Goldstone boson amplitude:

$$\delta\widetilde{\mathcal{N}}_s' = -\tfrac{1}{4}M_n^2(238c_\theta+19c_{3\theta}-c_{5\theta})\csc^4\theta\,, \tag{S28a}$$

$$\delta\widetilde{\mathcal{N}}_t' = \frac{M_n^2(99+8c_\theta+28c_{2\theta}-8c_{3\theta}+c_{4\theta})}{8(1-c_\theta)^2}\,, \tag{S28b}$$

$$\delta\widetilde{\mathcal{N}}_u' = -\frac{M_n^2(99-8c_\theta+28c_{2\theta}+8c_{3\theta}+c_{4\theta})}{8(1+c_\theta)^2}\,. \tag{S28c}$$

Using the double-copy formulas in Eqs.(18a)-(18b) together with the gauge-transformed numerators $(\mathcal{N}_j',\,\widetilde{\mathcal{N}}_j')$ in Eq.(19) and Eqs.(S27)-(S28), we construct the following four-point KK graviton amplitude and gravitational KK Goldstone amplitude at the LO and NLO:

$$\mathcal{M}_0(\text{DC}) = \widetilde{\mathcal{M}}_0(\text{DC}) = \frac{3\kappa^2}{128}\,s\,(7+c_{2\theta})^2\csc^2\theta\,, \tag{S29a}$$

$$\delta\mathcal{M}(\text{DC}) = -\frac{5\kappa^2M_n^2}{768}(1642+297c_{2\theta}+102c_{4\theta}+7c_{6\theta})\csc^4\theta\,, \tag{S29b}$$

$$\delta\widetilde{\mathcal{M}}(\text{DC}) = -\frac{\kappa^2M_n^2}{768}(6386+3837c_{2\theta}+30c_{4\theta}-13c_{6\theta})\csc^4\theta\,, \tag{S29c}$$

where we have set the conversion constant $c_0\!=\!-\kappa^2/(24g^2)$. The double-copy amplitudes of Eq.(S29a) provide the LO gravitational amplitudes (20) and the NLO gravitational amplitudes (21) in the main text. We can further compute the gravitational residual term of the GRET from the difference between the two NLO amplitudes (S29b) and (S29c):

$$\Delta\mathcal{M}(\text{DC}) = \delta\mathcal{M}(\text{DC}) - \delta\widetilde{\mathcal{M}}(\text{DC}) = -\kappa^2M_n^2\,(7+c_{2\theta})\,, \tag{S30}$$

which provides Eq.(22b) in the main text. We see that the above reconstructed residual term (S30) by the extended double-copy approach does give the same size of $\mathcal{O}(E^0M_n^2)$ and takes the same angular structure of $(1,\,c_{2\theta})$ as the original residual term (S24) although their numerical coefficients still differ. As discussed in the main test, it is impressive to note that Eq.(S30) also demonstrates a very precise cancellation between the angular structures $(1,\,c_{2\theta},\,c_{4\theta},\,c_{6\theta})\times\csc^4\theta$ of the NLO double-copied KK amplitudes (S29b)-(S29c) down to the substantially simpler angular structure $(1,\,c_{2\theta})$. This is the same kind of angular cancellations as what we found for the original NLO KK graviton and Goldstone amplitudes (S22b)-(S22c) and their difference (S24). This demonstrates that the above double-copied NLO KK amplitudes have captured the essential features of the original KK graviton (Goldstone) amplitudes at both the LO and NLO. We have presented the further improved NLO numerators (28)-(29) in the main text, which can realize the double-copied NLO KK amplitudes in full agreement with the original NLO KK graviton and Goldstone amplitudes (S22b)-(S22c). A further study based on the first principle approach of the KK string theory is recently presented in Ref. [4], which can realize the exact double-copy construction of the general N-point KK graviton scattering amplitudes at tree level.

Finally, for the sake of comparison, we also give the results of making the high energy expansion of M_n^2/s_0 and explain that within this expansion there is no generalized gauge transformation which could realize the Jacobi-conserving numerators for KK gauge boson (Goldstone) scattering amplitudes. For this, we express the elastic scattering amplitude $\mathcal{T}[4A_L^n]\equiv\mathcal{T}[A_L^{an}A_L^{bn}\to A_L^{cn}A_L^{dn}]$ and $\widetilde{\mathcal{T}}[4A_5^n]\equiv\widetilde{\mathcal{T}}[A_5^{an}A_5^{bn}\!\to\! A_5^{cn}A_5^{dn}]$ as follows:

$$\mathcal{T}[4A_L^n] = g^2\left(\frac{\mathcal{C}_s\mathcal{N}_s}{s_0}+\frac{\mathcal{C}_t\mathcal{N}_t}{t_0}+\frac{\mathcal{C}_u\mathcal{N}_u}{u_0}\right), \tag{S31a}$$

Table S1: Kinematic numerators of the LO and NLO scattering amplitudes (S31a)-(S31b) for KK longitudinal gauge bosons and KK Goldstones under the high energy expansion of M_n^2/s_0, where $(\mathcal{N}_j,\, \widetilde{\mathcal{N}}_j) = (\mathcal{N}_j^0,\, \widetilde{\mathcal{N}}_j^0) + (\delta\mathcal{N}_j,\, \delta\widetilde{\mathcal{N}}_j) = \mathcal{O}(E^2 M_n^0) + \mathcal{O}(E^0 M_n^2)$.

Numerators	$\mathcal{N}_s$	$\mathcal{N}_t$	$\mathcal{N}_u$	$\widetilde{\mathcal{N}}_s$	$\widetilde{\mathcal{N}}_t$	$\widetilde{\mathcal{N}}_u$	$\mathcal{N}_s - \widetilde{\mathcal{N}}_s$	$\mathcal{N}_t - \widetilde{\mathcal{N}}_t$	$\mathcal{N}_u - \widetilde{\mathcal{N}}_u$
$\mathcal{N}_j^0/s_0$	$-\frac{11c_\theta}{2}$	$\frac{-5+11c_\theta+4c_{2\theta}}{4}$	$\frac{5+11c_\theta-4c_{2\theta}}{4}$	$-\frac{3c_\theta}{2}$	$\frac{3(-3+c_\theta)}{4}$	$\frac{3(3+c_\theta)}{4}$	$-4c_\theta$	$-4c_\theta$	$-4c_\theta$
$\delta\mathcal{N}_j/M_n^2$	$4c_\theta$	$\frac{2(2-3c_\theta-2c_{2\theta}-c_{3\theta})}{1+c_\theta}$	$-\frac{2(2+3c_\theta-2c_{2\theta}+c_{3\theta})}{1-c_\theta}$	$4c_\theta$	$-\frac{8c_\theta}{1+c_\theta}$	$-\frac{8c_\theta}{1-c_\theta}$	0	$8s_\theta^2$	$-8s_\theta^2$

$$\widetilde{\mathcal{T}}[4A_5^n] = g^2\left(\frac{\mathcal{C}_s\widetilde{\mathcal{N}}_s}{s_0} + \frac{\mathcal{C}_t\widetilde{\mathcal{N}}_t}{t_0} + \frac{\mathcal{C}_u\widetilde{\mathcal{N}}_u}{u_0}\right). \tag{S31b}$$

We compute their numerators at the LO and NLO, $(\mathcal{N}_j,\, \widetilde{\mathcal{N}}_j) = (\mathcal{N}_j^0,\, \widetilde{\mathcal{N}}_j^0) + (\delta\mathcal{N}_j,\, \delta\widetilde{\mathcal{N}}_j) = \mathcal{O}(E^2 M_n^0) + \mathcal{O}(E^0 M_n^2)$, and present them in the following Tabel S1.

With these, we verify that the LO numerators of KK gauge boson (Goldstone) scattering amplitude satisfy the Jacobi identity:

$$\sum_j \mathcal{N}_j^0 = 0\,, \qquad \sum_j \widetilde{\mathcal{N}}_j^0 = 0\,, \tag{S32}$$

where $j \in (s,t,u)$. But, we find that the Jacobi identity is no longer obeyed by the NLO numerators:

$$\sum_j \delta\mathcal{N}_j = \sum_j \delta\widetilde{\mathcal{N}}_j = \chi \neq 0\,, \tag{S33a}$$

$$\chi = -2(7+c_{2\theta})c_\theta \csc^2\theta\, M_n^2\,, \tag{S33b}$$

We further note that the KK amplitudes (S31a)-(S31b) are invariant under the generalized gauge transformations for the kinematic numerators:

$$\mathcal{N}_j \to \mathcal{N}_j' = \mathcal{N}_j + \Delta \times s_{0j}\,, \qquad \widetilde{\mathcal{N}}_j \to \widetilde{\mathcal{N}}_j' = \widetilde{\mathcal{N}}_j + \widetilde{\Delta} \times s_{0j}\,. \tag{S34}$$

But, because of $\sum_j s_{0j} = 0$ [cf. Eq.(S4)], we deduce $\sum_j \delta\mathcal{N}_j' = \sum_j \delta\mathcal{N}_j \neq 0$ and $\sum_j \delta\widetilde{\mathcal{N}}_j' = \sum_j \delta\widetilde{\mathcal{N}}_j \neq 0$. Hence, under the expansion of M_n^2/s_0, it is impossible to obtain proper solutions of $\{\Delta\,, \widetilde{\Delta}\}$ which are supposed to ensure the gauge-transformed NLO numerators $(\delta\mathcal{N}_j',\, \delta\widetilde{\mathcal{N}}_j')$ to obey the kinematic Jacobi identity.

References

[1] Y. F. Hang and H.-J. He, Phys. Rev. D 105 (2022) 084005 [arXiv:2106.04568 [hep-th]].

[2] S. Weinberg, Physica 96A (1979) 327.

[3] R. S. Chivukula, D. Foren, K. A. Mohan, D. Sengupta, and E. H. Simmons, Phys. Rev. D 101 (2020) 075013 [arXiv:2002.12458 [hep-ph]].

[4] Y. Li, Y.-F. Hang, H.-J. He, and S. He, JHEP 02 (2022) 120 [arXiv:2111.12042 [hep-th]].
